# Recurrent hepatocellular carcinoma with osteoclast-like giant cells: a case report

**DOI:** 10.1186/s13256-022-03355-1

**Published:** 2022-04-01

**Authors:** Mone Tsukimoto, Kazushi Sugimoto, Ryuta Shigefuku, Ryosuke Sugimoto, Hiroto Yuasa, Katsunori Uchida, Norihiko Yamamoto

**Affiliations:** 1 Department of Gastroenterology and Hepatology, Graduate School of Medicine, Mie University, 2-174 Edobashi, Tsu, Mie 514-8507 Japan; 2 Department of Oncologic Pathology, Graduate School of Medicine, Mie University, 2-174 Edobashi, Tsu, Mie 514-8507 Japan; 3 Department of General medicine, Graduate School of Medicine, Mie University, 2-174 Edobashi, Tsu, Mie 514-8507 Japan

**Keywords:** Case report, Hepatocellular carcinoma, Osteoclast-like giant cells

## Abstract

**Background:**

Hepatocellular carcinoma with osteoclast-like giant cells is very rare and has an extremely poor prognosis. Here, we report a case of hepatocellular carcinoma with osteoclast-like giant cells that had a relatively better prognosis.

**Case presentation:**

A 70-year-old Japanese man with hepatitis B virus-related liver cirrhosis was admitted to our hospital for the treatment of recurrent hepatocellular carcinoma. At the age of 60 years, he was first diagnosed as having hepatocellular carcinoma in the right lobe (9 cm in diameter), and liver resection of segment 7/8 was performed. Histological findings showed well-differentiated hepatocellular carcinoma. Since then, imaging studies have been performed every 3 or 4 months. One year later, hepatocellular carcinoma recurred in the lateral segment, and radiofrequency ablation was performed. Nine years after the first presentation, hepatocellular carcinoma recurrences were detected in the caudate lobe and segment 5 by imaging studies. Surgical resection of the caudate lobe was performed, and ultrasonography-guided radiofrequency ablation was subsequently performed for the segment 5 tumor. The resected tumor was simple nodular, well-differentiated HCC; it measured 21 × 21 mm and contained many osteoclast-like giant cells. As neither vascular nor bile duct invasion was found, we believe that radical resection was achieved. Since then, the hepatocellular carcinoma has not recurred for over a year and a half.

**Conclusion:**

Hepatocellular carcinoma with osteoclast-like giant cells is very rare and the prognosis is extremely poor, but early detection can lead to a better clinical course.

## Background

Hepatocellular carcinoma (HCC) is one of the most prevalent cancers worldwide, especially in Asia and Africa. HCC arises from hepatocytes, which are the most predominant cell type of the liver. Most cases of HCC are secondary to chronic liver diseases, especially liver cirrhosis, due to hepatitis virus infection, alcoholic liver disease, or nonalcoholic steatohepatitis. HCC with osteoclast-like giant cells (OGCs) is very rare, and only a few cases have been previously reported. There have also been case reports showing that tumors arising from several other organs, including the ovary, pancreas, urinary tract, and thyroid gland, had OGCs; however, the origin of OGCs is still unclear. HCC with OGCs is reported to have an extremely poor prognosis. In most cases, patients are diagnosed at an advanced stage, and they die within a few months after diagnosis. Here, we report a case of HCC with OGCs that had a relatively longer recurrence-free survival.

## Case presentation

**Fig. 1:**
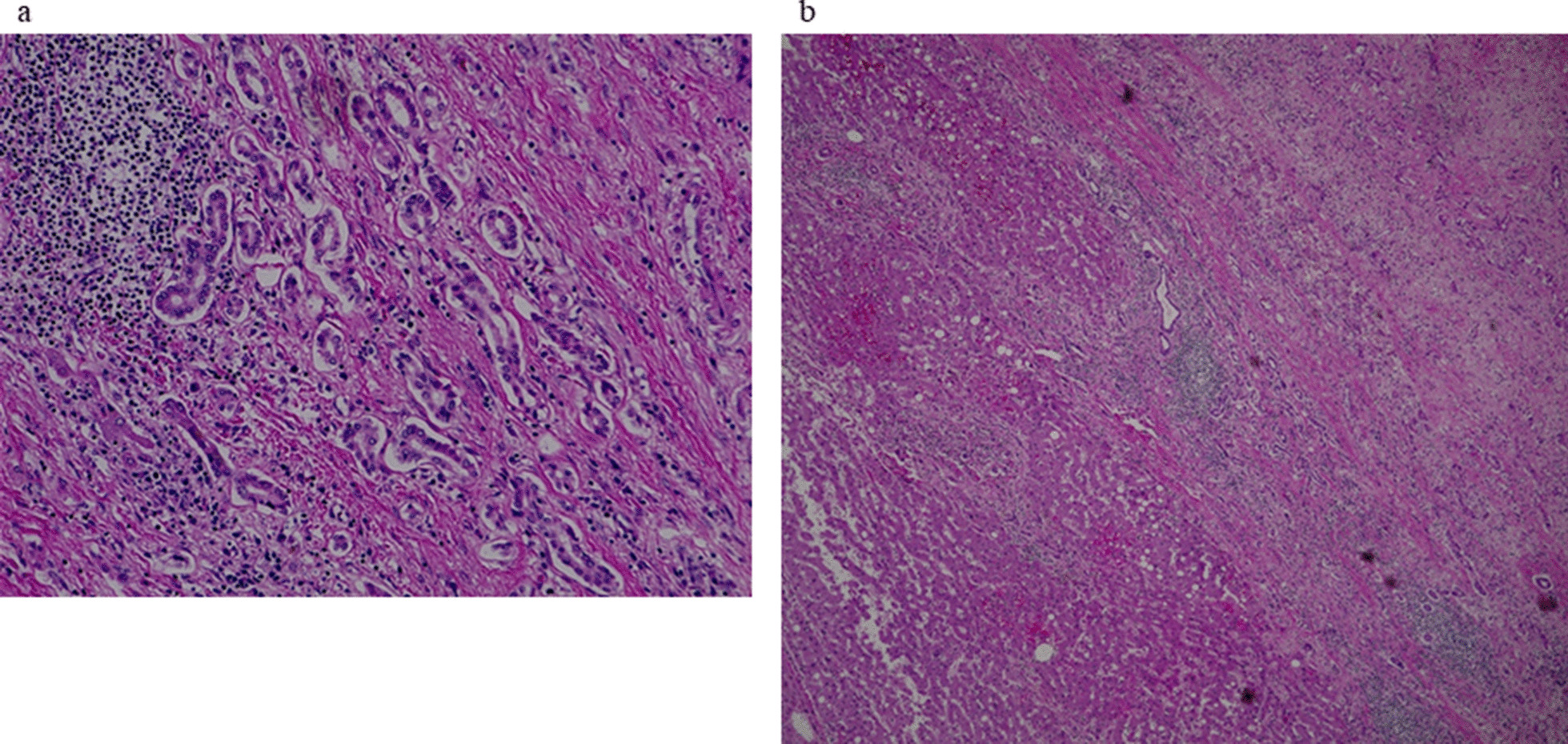
At the first presentation, the resected tumor was revealed to be well-differentiated hepatocellular carcinoma (**a**), and the surrounding tissue showed cirrhosis (**b**)

**Fig. 2:**
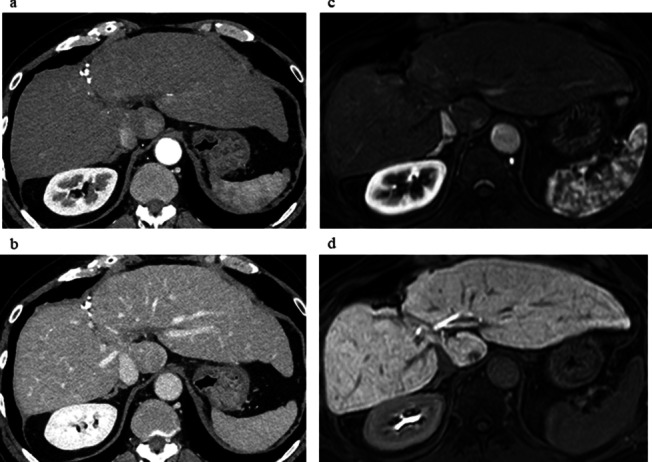
Preoperative imaging observations. Contrast-enhanced CT reveals the tumor in the caudate lobe. The tumor was slightly enhanced at the arterial phase (**a**), and washed out at the venous phase (**b**). MRI contrasted with gadoxetate sodium also shows the tumor in the caudate lobe (arterial phase (**c**) and hepatobiliary phase (**d**))

A 70-year-old Japanese man was admitted to our hospital for the treatment of recurrent liver tumors. He had taken medications for hypertension, hyperuricemia, and depression since he was around 60 years old. At the age of 50 years, he was diagnosed as having liver cirrhosis due to chronic hepatitis B virus (HBV) infection. In 2010, at the age of 60 years, he was diagnosed for the first time as having HCC in the right lobe of the liver, and was admitted to our hospital. The serum α-fetoprotein (AFP) level was 110 ng/dl (reference value < 10 ng/dl), and the des-γ-carboxy prothrombin (DCP) level was 8926 mAU/ml (reference value < 40 mAU/ml). Liver resection of segment 7/8 was performed; the pathological diagnosis of the resected tissue was well-differentiated HCC, and the surrounding tissue showed cirrhosis (Fig. 1). After surgical resection, both the serum AFP and DCP levels decreased, to 10 ng/ml and 16 AU/ml, respectively, and entecavir was subsequently started. One year later, HCC recurrence was found in the lateral segment of the liver, and radiofrequency ablation (RFA) was performed. Neither the serum AFP level (7 ng/dl) nor the DCP level (25 mAU/ml) was increased at the time of this recurrence. In 2017, he underwent surgical resection of the right lung for lung cancer, and the histological diagnosis was adenocarcinoma originating from lung tissue. In June 2019, a tumor lesion with a diameter of 14 mm in the caudate lobe was found by magnetic resonance imaging (MRI) contrasted with gadoxetate sodium (Fig. 2), and he was again admitted to our hospital.

On admission, he had no symptoms. Laboratory tests showed a serum aspartate aminotransferase level of 29 U/l, alanine aminotransferase level of 24 U/l, alkaline phosphatase level of 149 U/l, lactate dehydrogenase level of 188 U/l, γ-glutamyl transpeptidase level of 42 U/l, serum hepatitis B surface antigen level of 69.81 IU/ml, serum AFP level of 6 ng/dl, DCP level of 37 mAU/ml, and HBV DNA level lower than 1.3 log IU/ml. Contrast-enhanced computed tomography (CT) showed that the tumor in the caudate lobe was enhanced at the arterial phase, and the contrast medium was washed out at the hepatocyte phase. In addition, another tumor with the same characteristics was found in segment 5. Abdominal angiography with CT arterioportography and CT hepatic arteriography (CTHA) was performed for the definitive diagnosis. Both tumors appeared as perfusion defects by CT arterioportography, and hypervascular lesions by early-phase CTHA, and lesions with rim enhancements on delayed-phase CTHA. The diagnoses of recurrent HCC for both tumors were made on the basis of these image study findings. Surgical resection of the caudate lobe was performed, and ultrasonography-guided RFA was subsequently performed for the segment 5 HCC.

**Fig. 3:**
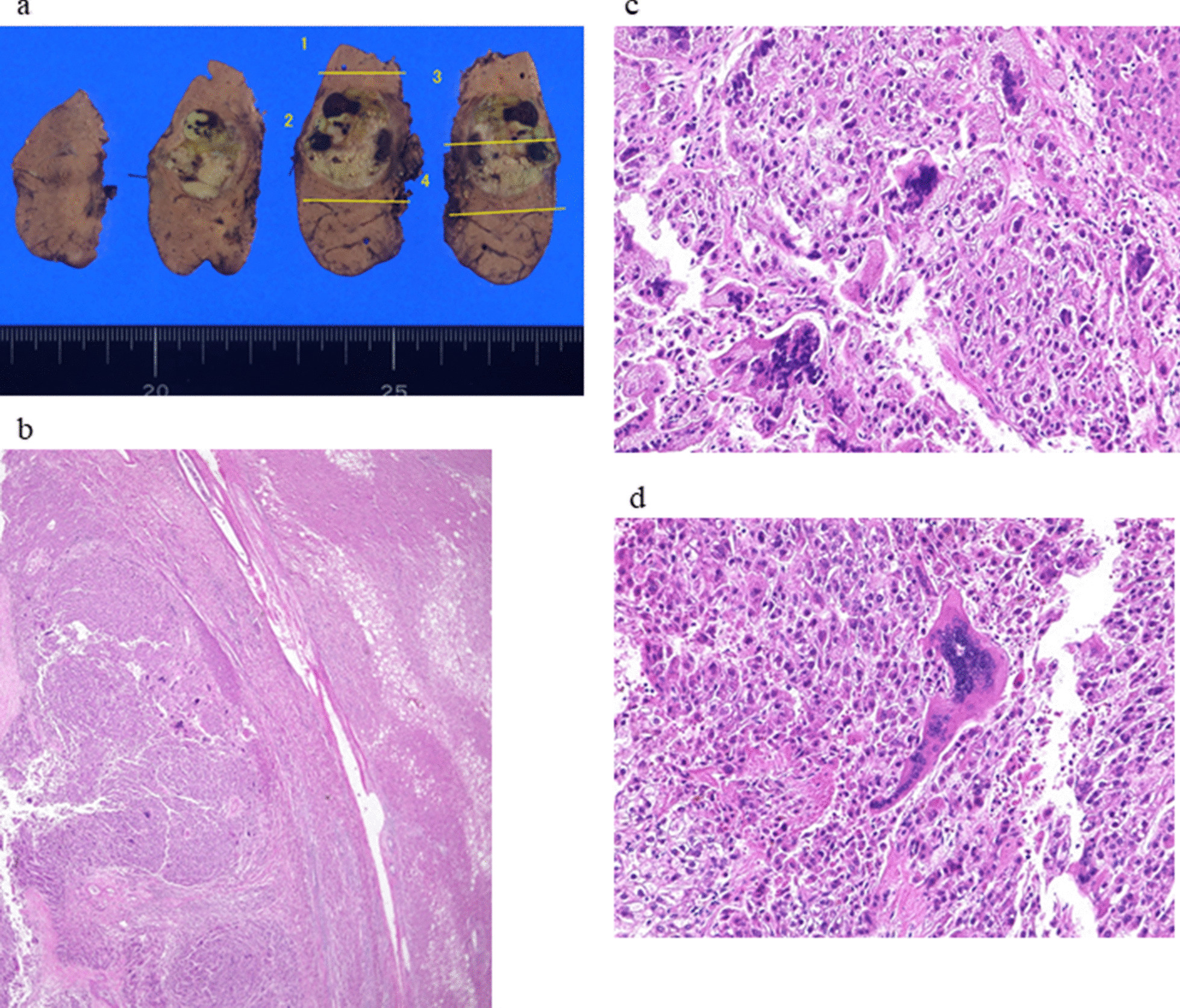
Macroscopic findings of the excised hepatocellular carcinoma tissue (**a**). Microscopic findings of osteoclast-like giant cell tumor (OGCT) and hepatocellular carcinoma (HCC) components (HE; low-power field view) (**b**). High-power field views of OGCT and HCC (**c** and **d**)

The resected tumor was a simple nodular tumor that measured 21 × 21 mm. Histologically, it was diagnosed as HCC containing well-differentiated and moderately differentiated components. In addition, many giant cells with multiple nuclei, which were not present in the first resected sample, were found in the tumor tissues, and they were diagnosed as OGCs (Fig. 3). Neither vascular nor bile duct invasion was detected. According to these findings, we believe radical resection was achieved. Ever since these therapeutic procedures were completed, the patient has been followed up at the out-patient clinic, and HCC has not recurred for more than a year and a half.

## Discussion

The prognosis of HCC has significantly improved recently owing to the evolution of diagnostic methods (such as tumor markers and imaging studies) and therapeutic methods (such as chemoembolization, RFA, and molecular targeted drugs). However, it has been reported that patients with HCC containing OGCs still have extremely shorter survival periods than patients with normal HCC. The present case is a rare one in which the patient had recurrence-free survival for more than a year and a half after the second surgical resection for recurrent HCC even though the histological study revealed OGCs in the resected recurrent HCC.

Cases of HCC with OGCs were first reported in 1984, and to the best of our knowledge, in total, ten cases have been reported in the English literature. All ten cases are reviewed and summarized in Table 1 [1–10]. Five patients were male and five patients were female. Their ages ranged from 37 to 76 years with a mean of 62 years, and seven patients were over 60 years of age. Five patients had a cirrhotic liver, and three cases had sarcoid-like changes. The diameter of the tumor at the time of discovery ranged from 6 to 12 cm. According to these reports, HCC with OGCs grows much more rapidly than normal HCC. In addition, many cases already had distant metastases and/or peritoneal dissemination, and radical resections were difficult. Even in cases in which surgical resection was performed, tumor recurrence occurred immediately after surgery, and the patients died soon thereafter. In fact, five of ten patients died less than 3 months after diagnosis.

**Table 1:** All ten cases of HCC with OGCs

No.	Author	Year	Age (years)	Sex	Diagnosis	Cirrhosis	Prognosis
1	Kuwano	1984	54	Male	HCC with OGC	Yes	Death (42 days)
2	Hood	1990	37	Female	Hepatic giant cell carcinoma	No	Death (3 months)
3	McCluggage	1997	71	Male	HCC with OGC	Yes	Death (1 month)
4	Sasaki	1997	42	Male	Sarcomatoid HCC with OGC	Yes	Death (28 days)
5	Ikeda	2003	76	Male	HCC with OGC	Yes	Death (5 months)
6	Rudloff	2005	61	Female	HCC and OGC	No	Death (3 months)
7	Tanahashi	2009	74	Female	Combined HCC and OGC	No	Death (4 months)
8	Lee K B	2014	64	Male	Sarcomatoid HCC with OGC	Yes	Recurrence (2 months)
9	Dioscoridi	2015	74	Female	HCC with OGC	No	Death (4 months)
10	Dahm	2015	68	Male	Sarcomatoid HCC with OGC	No	Recurrence (3 months)

*HCC* hepatocellular carcinoma,* OGC* Osteoclast-like giant cell

In general, HCC with OGCs comprises mononuclear cells, OGCs, and HCC [1, 4–7]. The origin of OGCs remains unclear and controversial. However, in recent reports, OGCs have generally been considered to be reactive histiocytes rather than true malignant tumor cells because, in many cases, OGCs were positive for the immunostaining of histiocytic marker CD68 and mesenchymal marker vimentin, but not of epithelial markers, such as cytokeratin [5–8]. In addition, a study by Tanahashi on the Ki-67 index reported that Ki-67 positivity was 10% for HCC, 60% for spindle-shaped cells, and 0% for giant cells [7], indicating that OGCs are essentially not tumor cells, although HCC with OGCs is very aggressive.

The current case suggests several points. Firstly, even though the patient started to take a nucleot(s)ide analog (NA) for HBV at the time of the first liver resection for HCC and, thereafter, the serum alanine aminotransferase level had remained below the upper limit and the serum HBV DNA level had been suppressed below the detection level (1.3 log IU/ml), HCC recurred 10 years later. Although an inhibitory effect of NA treatment against development of HCC has been reported in patients with chronic hepatis B [11], it is suggested that the suppression of HBV proliferation and liver inflammation by NA does not mean that the carcinogenesis has been completely eliminated in cirrhotic liver, as in this case.

Recently, attention has been paid to the relationship between HBV core-related antigen and liver carcinogenesis [12]. Our case suggests that NA may not exert a carcinogenic-inhibitory effect, and the HBV core-related antigen level remained high even when the blood HBV DNA amount became undetectable. Furthermore, the serum AFP and DCP levels did not increase at recurrence even though they were increased at the first HCC occurrence, and pathological findings showed that OGCs were not included in the first resected specimen. These findings suggest that the HCC at recurrence had different properties, and the cause of carcinogenesis may have differed from that of the first HCC.

Secondly, our case had a longer survival period than previously reported cases of HCC with OGCs. In addition, imaging studies, including abdominal MRI and positron emission tomography/CT scans with [^18^F]-fluorodeoxyglucose, showed no evidence of recurrence or metastasis for more than a year and a half after the second resection of HCC. We think the reason for the better outcome in our case is that very strict surveillance of HCC was performed by imaging studies on a regular basis; therefore, the HCCs were detected earlier when the diameters were relatively smaller for both the primary and recurrent lesions, and the tumors did not invade the vasculature or bile ducts, indicating that curative resections were obtained both times. In contrast, most of the previous reports were of the first HCC occurrence, and the patients did not undergo regular surveillance for HCC, so tumors were not found until they had grown bigger in size.

## Conclusion

Our case suggests that even in cases of HCC with OGCs, which were originally thought to have extremely poor prognoses, satisfactory survival can be expected if they can be detected and treated at an early stage. However, continued careful follow-up is needed for this patient with the use of imaging modalities, such as dynamic CT and/or MRI.

## Data Availability

The dataset supporting the conclusions of this article is included within the article.

## Abbreviations

HCC: Hepatocellular carcinoma
OGCs: Osteoclast-like giant cells
HBV: Hepatitis B virus
AFP: α-Fetoprotein
DCP: Des-γ-carboxy prothrombin
RFA: Radiofrequency ablation
MRI: Magnetic resonance imaging
CT: Computed tomography
CTHA: CT during arterioportography
